# Quality of life and participation restrictions, a study in elderly

**DOI:** 10.1590/S1808-86942011000500016

**Published:** 2015-10-22

**Authors:** Ruth Magalhães, Maria Cecilia Martinelli Iório

**Affiliations:** 1 Master's degree in science, Sao Paulo Federal University (Universidade Federal de São Paulo, Unifesp). Speech therapist, Auditory Health Clinic, Santo Amaro Holy House of Mercy (Santa Casa de Misericórdia de Santo Amaro); 2 Assistant professor, Sao Paulo Federal University (Universidade Federal de São Paulo). Head of the Phonoaudiology Department

**Keywords:** health of the elderly, hearing aids, hearing loss, quality of life, questionnaires

## Abstract

**Abstract:**

According to the IBGE, Brazil had 21 million elderly persons in 2008.

**Objective:**

To study the effects of speech therapy - fitting of hearing aids - on the quality of life of elderly persons and restriction of participation according to sex and age.

**Material and Method:**

50 elderly subjects, 23 females and 27 males, were allocated to Group 1 and Group 2, and were assessed with questionnaires (HHIE and SF 36) before and one year after fitting hearing aids; subjects were monitored every two months.

**Results:**

The HHIE Social and Emotional Scale was significant with regards to sex and age after fitting hearing aids. The SF 36 results after fitting hearing aids were significant in six of eight test aspects. Two test aspects were not significant after fitting hearing aids; pain, however, was significant in the elderly group 2.

**Conclusion:**

There is little awareness of participation restrictions after the HHIE intervention. There is improvement in quality of life after rehabilitation. Retrospective study.

## INTRODUCTION

One of the most devastating and incapacitating deficiencies of old age is presbiacusis. Hearing loss causes difficulties in understanding speech, affects communication, and compromises family and social life. Thus, auditory loss in the elderly affects not only hearing but also has marked psychosocial implications^1^.

According to the Pan-American Health Organization (PAHO), healthy and active aging implies valuing autonomy and preserving physical and psychic autonomy, among other factors, and preventing functional losses or reducing their negative effects^2^.

In the Brazilian Unified Health System (Sistema Unico de Saude, or SUS), it is considered essential to assure full care to the elderly population and to emphasize healthy and active aging (No. 2,528, 19 October 2006). It is thus necessary to learn about the Brazilian elderly population; according to the Brazilian Geography and Statistical Institute (Instituto Brasileiro de Geografia e Estatística, or IBGE) there are nearly 21 million elderly people in Brazil^3^.

The national policy for the elderly aims to assure social rights for this population group by promoting autonomy and effective participation in society with the purpose of maintaining quality of life. According to the IBGE, if the fertility and longevity trends continue in Brazil, the number of elderly people is estimated to be over 30 million in the next 20 years, which comprises nearly 13% of the population.

Living longer may imply in a life of physical incapacity and dependence. There is a further challenge for healthcare professionals working with elderly people: to assess quality of life rather than only to measure aging, and the impact of treatments and public policies for this age group^4^.

In this context, healthcare professionals need to specifically study this age group. Questionnaires have been used as evaluation tools to measure the difficulties of elderly individuals and their perception of these handicaps. One of these questionnaires is the Hearing Handicap Inventory for the Elderly; it aims to assess the impact of hearing loss on the emotional and social realities of elderly subjects. Another such questionnaire is the Short Form Health Survey - SF 36, which aims to assess health and quality of life; it consists of two parts: the first investigates the health status (questions about physical mobility, pain, sleep, energy, social isolation, and emotional reactions) and the second evaluates the impact of disease on the patient's daily life.

A few questions were subsequently raised: what is the impact of hearing loss on the quality of life of elderly individuals? Do men or women have more perception of restrictions in daily life? Which age group has the most compromised quality of life?

It is evident that a protocol for assessing the quality of life of elderly individuals and their opinion about issues they consider important is needed.

Therefore, the purpose of this study was to investigate the effects of phonoaudiological interventions (fitting hearing aids) in relation to restricted participation in daily life activities and quality of life of elderly individuals; the variables were sex and age.

## MATERIAL AND METHODS

The institutional review board assessed and approved the study design (no. CEP 0913/08). Patients that agreed to participate signed a free informed consent form (based on the Resolution 196/96 on guidelines about research in human beings).

The following inclusion criteria were defined: age over 60 years, up to severe bilateral symmetrical sensorineural hearing loss, speech recognition score over 50%, indication for adapting binaural hearing aids, no evidence of other conditions, and being literate.

The sample comprised 50 elderly subjects - 23 female (46%) and 27 male (54%) - aged 60 years or above. The subjects were allocated to two age groups, as follows: Group 1 (G1), which consisted of 24 subjects (48%) aged from 60 to 74 years, of which 11 were female and 13 were male, and Group 2 (G2), which consisted of 26 subjects (52%) aged 75 years and above, of which 12 were female and 14 were male.

Audiological testing consisted of pure tone audiometry, play audiometry, and immittance testing, which were done at our Clinic by expert staff speech therapists.

The self-assessment questionnaires used in this study were the Hearing Handicap Inventory for the Elderly (HHIE) and the Medical Outcome Study 36-Item Short Form Health Survey (SF 36).

The HHIE questionnaire was developed to assess the psychosocial, emotional, and social consequences of hearing loss in elderly patients. It was originally developed in English language and adapted to Portuguese by Wieselberg (1997). We used the pencil and paper application technique.^5^

The HHIE consists of 25 questions, of which 13 explore the emotional and 12 the social/situational consequences of hearing loss. Restrictions to participate are classified according to a scoring system in which four points are given to the answer “yes”, two points to the answer “sometimes”, and zero points to the answer “no”. A higher score means that an individual is more aware of his restrictions to participate - hearing loss imposes more auditory and non-auditory difficulties. The scores are distributed as follows: no perception of a handicap = 0 to 16; mild to moderate perception of a handicap = 18 to 42; and severe/significant perception of a handicap = > 42 points.

The Medical Outcome Study 36-Item Short Form Health Survey (SF 36) was first developed and then translated for the Brazilian population^6^,^7^. It is a generic tool that is used to assess the quality of life of patients; it consists of 11 questions and 36 items on different topics, as follows: functional ability, physical aspects, emotional aspects, intensity of pain, general health status, vitality, social aspects, and mental health. The application technique was paper and pencil.

Eight aspects were evaluated, as follows: functional capacity - 10 items; physical aspects and vitality - four items; emotional aspects - three items; pain and social aspects - two aspects; general health status and mental health - five items. Each subject is scored in each aspect, which is then consolidated in a 0 to 100 scale where 0 is the worst score and 100 is the best; thus, higher scores indicate better quality of life.

Seven meetings with two-month intervals between each were scheduled to monitor the patients as they adapted to hearing aids. During these meetings patients were oriented about the use, care, and handling of hearing aids, as well as communication strategies.

1^st^ meeting - The elderly subjects answered the printed SF 36 and HHIE questionnaires. After this meeting and during August 2008, retroauricular bilateral hearing aids were fitted; all were of the same brand and type, and had been selected beforehand by the institution staff.

2^nd^ meeting - Elderly subjects at this point were adapted to their hearing aids and could clear up any doubts about their use, handling, and care. In this meeting, each subject was given the opportunity to describe his or her difficulties and victories with using hearing aids up to this point.

3^rd^ meeting - Subjects were given instructions about how to use telephones. Doubts about handling and care of hearing aids were discussed.

4^th^ meeting - In this meeting each subject was given a dehumidifier and a device for cleaning the molds. Verbal and practical instructions were given about how to use these materials, and an explanatory folder was also handed out.

5^th^ meeting - Elderly subjects now have used the hearing aids for over six months; but instructions about their use, care, and handling were repeated. The group could be seen to interact; those that had adapted better helped other with difficulties.

6^th^ meeting - Each subject was given a key ring that tested the hearing aid battery; verbal and practical instructions about how to use this device were given; an explanatory folder was also handed out.

7^th^ meeting - In this last meeting, after about one year of bilateral hearing aid use, all elderly subjects were answered the SF 36 and HHIE questionnaires.

## STATISTICS

The sample was characterized according to sex and age. Tables containing descriptive statistical values for the HHIE and SF 36 scores were made for each period, sex, and age group.

Analysis of variance with repeated measures was applied to compare the mean scores of questionnaires (two evaluations), sex, and age groups^8^. After this analysis, the means of differences between both tests (pre and post) were estimated, and 95% confidence intervals were made for these means^9^.

The statistics software Minitab version 15 and the SPSS version 11 were used in the statistical analysis. The significance level for each hypothesis was 0.05, and the significant p-values were marked with an asterisk (*).

## RESULTS

The study sample comprised 50 elderly subjects, 23 female (46%) and 27 male (54%). There were 24 subjects aged from 60 to 74 years (48%) and 26 subjects aged over 75 years (52%).

Descriptive statistics were made for the HHIE questionnaire scores before (pre) and after (post) the intervention (Table 1).

**Table 1:** Descriptive statistics for total HHIE scores before and after the intervention.

Variable	N	Mean	Standard deviation	Minimum	Median	Maximum
Total pre	50	32.9	5.9	20	34	42
Total post	50	8.8	5.1	0	8	22

Pré x Pós - *p* = 0, 000*

Tables 2 and 3 show the questionnaire scores for the emotional and social scales.

**Table 2:** Analysis of variance and descriptive statistics for the emotional scale scores in the HHIE, according to sex and age group.

Sex	Age group	Period	N	Mean	Standard deviation	Minimum	Median	Maximum
		Pre	11	12,7	3.6	8	12	18
	60 to 74	Post	11	4.0	2.0	0	4	6
		Pre	12	15.2	4.0	10	16	20
Female	75 or +	Post	12	3.8	3.8	0	3	12
		Pre	23	14.0	3.9	8	14	20
	Total	Post	23	3.9	3.0	0	4	12
		Pre	13	15.8	3.8	10	18	20
	60 to 74	Post	13	4.9	2.5	0	6	8
		Pre	14	17.6	2.7	12	18	20
Male	75 or +	Post	14	3.4	3.1	0	2	12
		Pre	27	16.7	3.3	10	18	20
	Total	Post	27	4.1	2.9	0	4	12
		Pre	24	14.4	4.0	8	14	20
	60 to 74	Post	24	4.5	2.3	0	4	8
		Pre	26	16.5	3.5	10	17	20
Total	75 or +	Post	26	3.6	3.3	0	2	12
		Pre	50	15.5	3.8	8	16	20
	Total	Post	50	4.0	2.9	0	4	12

Analysis of variance

Pre x Post x Sex → *p* = 0.021*

Female x Pre x Post → *p* = 0.000*

Male x Pre x Post → *p* = 0.000*

Pre x Female x Male → *p* = 0.003*

Pre x Post x Age group → *p* = 0.008*

G1 (60 to 74 years) x Pre x Post → *p* = 0.000*

G2 (75 years or +) x Pre x Post → *p* = 0.000*

Pre x G1 (60 to 74 years) x G2 (75 years or +) → *p* = 0.026*

**Table 3:** Analysis of variance and descriptive statistics for HHIE scores in the social/situational scale according to sex and age group.

Sex	Age group	Period	N	Mean	Standard deviation	Minimum	Median	Maximum
		Pre	11	14.2	4.2	8	16	20
	60 to 74	Post	11	4.4	3.9	0	4	10
		Pre	12	17.0	2.6	12	16	20
Female	75 or+	Post	12	4.8	2.8	2	4	10
		Pre	23	15.7	3.7	8	16	20
	Total	Post	23	4.6	3.3	0	4	10
		Pre	13	18.5	2.0	14	20	20
	60 to 74	Post	13	5.1	3.0	0	6	8
		Pre	14	19.3	1.5	16	20	20
Male	75 or+	Post	14	4.7	2.8	2	4	12
		Pre	27	18.9	1.8	14	20	20
	Total	Post	27	4.9	2.8	0	6	12
		Pre	24	16.5	3.8	8	18	20
	60 to 74	Post	24	4.8	3.4	0	6	10
		Pre	26	18.2	2.4	12	20	20
Total	75 or+	Post	26	4.8	2.7	2	4	12
		Pre	50	17.4	3.2	8	18	20
	Total	Post	50	4.8	3.0	0	4	12

Analysis of variance

Pre x Post x Sex → *p* = 0.004*

Female x Pre x Post → *p* = 0.000*

Male x Pre x Post → *p* = 0.000*

Pre x Female x Male → *p* = 0.000*

Table 4, Table 5, Table 6, Table 7, Table 8, Table 9, Table 10 present descriptive statistics and analysis of variance values for seven of the eight aspects evaluated in the SF 36 questionnaire before and after the intervention, according to sex and age group. We chose not to show the descriptive statistics for the general health status aspect in the SF 36 questionnaire, as no statistically significant differences were observed in pre and post-intervention scores.

**Table 4:** Analysis of variance and descriptive statistics for the functional ability aspect according to sex and age group.

Sex	Faixa etária	Período	N	Média	Desvio padrão	Mínimo	Mediana	Máximo
		Pre	11	53.2	20.4	30	50	90
	60 to 74	Post	11	59.1	13.9	40	60	90
		Pre	12	49.2	25.5	0	47.5	80
Female	75 or+	Post	12	57.5	25.2	5	50	95
		Pre	23	51.1	22.8	0	50	90
	Total	Post	23	58.3	20.1	5	50	95
		Pre	13	49.6	11.4	35	50	70
	60 to 74	Post	13	56.9	19.7	30	50	100
		Pre	14	49.6	25.9	15	47.5	90
Male	75 or+	Post	14	54.3	22.5	15	50	90
		Pre	27	49.6	19.9	15	50	90
	Total	Post	27	55.6	20.9	15	50	100
		Pre	24	51.3	15.9	30	50	90
	60 to 74	Post	24	57.9	17.0	30	50	100
		Pre	26	49.4	25.2	0	47.5	90
Total	75 or+	Post	26	55.8	23.4	5	50	95
		Pre	50	50.3	21.1	0	50	90
	Total	Post	50	56.8	20.4	5	50	100

Analysis of variance

Pre x Post → *p* = 0.013*

**Table 5:** Analysis of variance and descriptive statistics for the physical status aspect according to sex and age group.

Sex	Age group	Period	N	Mean	Standard deviation	Minimum	Median	Maximum
		Pre	11	25.0	29.6	0	25	75
	60 to 74	Post	11	40.9	32.2	0	50	100
		Pre	12	25.7	18.6	0	25	50
Female	75 or+	Post	12	45.8	33.4	0	50	100
		Pre	23	25.4	23.9	0	25	75
	Total	Post	23	43.5	32.2	0	50	100
		Pre	13	21.8	38.1	0	0	100
	60 to 74	Post	13	46.2	38.0	0	50	100
		Pre	14	23.2	30.2	0	12.5	100
Male	75 or+	Post	14	46.4	35.2	0	50	100
		Pre	27	22.5	33.6	0	0	100
	Total	Post	27	46.3	35.8	0	50	100
		Pre	24	23.3	33.8	0	0	100
	60 to 74	Post	24	43.8	34.8	0	50	100
		Pre	26	24.4	25.0	0	25	100
Total	75 or+	Post	26	46.2	33.7	0	50	100
		Pre	50	23.8	29.3	0	25	100
	Total	Post	50	45.0	33.9	0	50	100

Analysis of variance

Pre x Post → *p* = 0.000*

**Table 6:** Analysis of variance and descriptive statistics of the pain aspect according to sex and age group.

Sex	Age group	Period	N	Mean	Standard deviation	Minimum	Median	Maximum
	Pre	11	54.8	27.9	22	51	100	
	60 to 74	Post	11	57.5	32.2	12	51	100
		Pre	12	60.8	23.8	31	51.5	100
Female	75 or+	Post	12	62.8	17.0	42	56.5	100
		Pre	23	58.0	25.4	22	51	100
	Total	Post	23	60.2	25.0	12	51	100
		Pre	13	48.6	22.2	12	51	84
	60 to 74	Post	13	52.8	23.6	12	51	100
		Pre1	45	7.4	18.8	11	56.5	100
Male	75 or+	Post	14	59.6	13.6	51	52	100
		Pre	27	53.2	20.6	11	52	100
	Total	Post	27	56.3	19.0	12	51	100
		Pre	24	51.5	24.6	12	51	100
	60 to 74	Post	24	54.9	27.4	12	51	100
		Pre	26	59.0	20.9	11	52	100
Total	75 or+	Post	26	61.0	15.1	42	52	100
		Pre	50	55.4	22.8	11	51	100
	Total	Post	50	58.1	21.8	12	51	100

Analysis of variance

G1 (60 to 74 years) x G2 (75 years or +) → *p* = 0.017*

**Table 7:** Analysis of variance and descriptive statistics for the vitality aspect according to sex and age group.

Sex	Age group	Period	N	Mean	Standard deviation	Minimum	Median	Maximum
		Pre	11	42.5	17.9	20	40	70
	60 to 74	Post	11	51.4	23.6	15	65	90
		Pre	12	44.7	20.7	20	40	90
Female	75 or+	Post	12	50.1	26.8	0	52.5	90
		Pre	23	43.6	19.0	20	40	90
	Total	Post	23	50.7	24.7	0	60	90
		Pre	13	46.2	20.6	5	55	75
	60 to 74	Post	13	63.1	17.7	30	65	90
		Pre	14	49.6	17.8	15	50	75
Male	75 or+	Post	14	54.6	15.9	25	60	80
		Pre	27	48.0	18.9	5	50	75
	Total	Post	27	58.7	17.0	25	65	90
		Pre	24	44.5	19.1	5	48.5	75
	60 to 74	Post	24	57.7	21.0	15	65	90
		Pre	26	47.3	19.0	15	47.5	90
Total	75 or+	Post	26	52.5	21.3	0	60	90
		Pre	50	46.0	18.9	5	47.5	90
	Total	Post	50	55.0	21.1	0	65	90

Analysis of variance

Pre x Post → *p* = 0.006*

**Table 8:** Analysis of variance and descriptive statistics for the social aspect according to sex and age group.

Sexo	Age group	Period	N	Mean	Standard deviation	Minimum	Median	Maximum
		Pre	11	43.1	21.1	0	50	75
	60 to 74	Post	11	70.5	29.7	12.5	87.5	100
		Pre	12	59.3	24.0	25	50	100
Female	75 or+	Post	12	67.7	25.3	25	68.75	100
		Pre	23	51.6	23.6	0	50	100
	Total	Post	23	69.0	26.9	12.5	75	100
		Pre	13	52.9	17.8	25	50	75
	60 to 74	Post	13	78.8	18.7	25	87.5	100
		Pre	14	59.5	25.8	0	56.25	100
Male	75 or+	Post	14	65.2	30.3	0	75	100
		Pre	27	56.3	22.2	0	50	100
	Total	Post	27	71.8	25.8	0	87.5	100
		Pre	24	48.4	19.6	0	50	75
	60 to 74	Post	24	75.0	24.2	12.5	87.5	100
		Pre	26	59.4	24.5	0	50	100
Total	75 or+	Post	26	66.3	27.6	0	68.75	100
		Pre	50	54.1	22.7	0	50	100
	Total	Post	50	70.5	26.1	0	87.5	100

Analysis of variance

Pre x Post → *p* = 0.000*

Age group x Pre x Post → *p* = 0.013*

G1 (60 a 74 years) x Pre x Post → *p* = 0.000*

**Table 9:** Analysis of variance and descriptive statistics for the emotional aspect according to sex and age group.

Sex	Age group	Period	N	Mean	Standard deviation	Minimum	Median	Maximum
		Pre	11	18.2	27.3	0	0	66.6
	60 to 74	Post	11	42.4	36.8	0	66.6	100
		Pre	12	38.9	42.2	0	33.3	100
Female	75 or+	Post	12	58.3	37.9	0	66.6	100
		Pre	23	29.0	36.6	0	0	100
	Total	Post	23	50.7	37.4	0	66.6	100
		Pre	13	30.8	44.0	0	0	100
	60 to 74	Post	13	61.5	35.6	0	66.6	100
		Pre	14	30.9	30.5	0	33.3	100
Male	75 or+	Post	14	52.4	40.7	0	66.6	100
		Pre	27	30.8	36.9	0	33.3	100
	Total	Post	27	56.8	37.9	0	66.6	100
		Pre	24	25.0	37.1	0	0	100
	60 to 74	Post	24	52.7	36.7	0	66.6	100
		Pre	26	34.6	35.9	0	33.3	100
Total	75 or+	Post	26	55.1	38.8	0	66.6	100
		Pre	50	30.0	36.4	0	16.65	100
	Total	Post	50	54.0	37.4	0	66.6	100

Analysis of variance

Pre x Post → *p* = 0.000*

Sex x Age group → *p* = 0.049*

Female x Age group → *p* = 0.050*

**Table 10:** Analysis of variance and descriptive statistics for the mental health aspect according to sex and age group.

Sex	Age group	Period	N	Mean	Standard deviation	Minimum	Median	Maximum
		Pre	11	18.2	27.3	0	0	66.6
	60 to 74	Post	11	42.4	36.8	0	66.6	100
		Pre	12	38.9	42.2	0	33.3	100
Female	75 or+	Post	12	58.3	37.9	0	66.6	100
		Pre	23	29.0	36.6	0	0	100
	Total	Post	23	50.7	37.4	0	66.6	100
		Pre	13	30.8	44.0	0	0	100
	60 to 74	Post	13	61.5	35.6	0	66.6	100
		Pre	14	30.9	30.5	0	33.3	100
Male	75 or+	Post	14	52.4	40.7	0	66.6	100
		Pre	27	30.8	36.9	0	33.3	100
	Total	Post	27	56.8	37.9	0	66.6	100
		Pre	24	25.0	37.1	0	0	100
	60 to 74	Post	24	52.7	36.7	0	66.6	100
		Pre	26	34.6	35.9	0	33.3	100
Total	75 or+	Post	26	55.1	38.8	0	66.6	100
		Pre	50	30.0	36.4	0	16.65	100
	Total	Post	50	54.0	37.4	0	66.6	100

Analysis of variance

Pre x Post → *p* = 0.004*

## DISCUSSION

The increasing elderly population in Brazil requires healthcare programs to provide adequate quality of life for this age group.

The impact of auditory loss on the quality of life of individuals may be measured by the configuration and degree of hearing loss, as well as the age of patients when this condition arises. Thus, decreased participation in activities of daily life due to hearing loss compromises social and professional performance, and therefore quality of life.

The mean values of the total HHIE score were 32.9% (pre-intervention) and 8.8% (post-intervention), which was a significant improvement. These results are compatible with the self-awareness of moderate participation restriction in the pre-adaptation period and non-awareness in the post-adaptation period.

Authors in several studies that applied the HHIE questionnaire have reported that elderly individuals need to be included in hearing rehabilitation programs to minimize the psychosocial reactions to hearing loss. These authors have also found that elderly subjects show effectively fewer restrictions to participation after taking part in hearing rehabilitation programs. The importance of hearing aid fitting is also underlined^10^.

A separate analysis of the emotional and social/situational scores revealed that the mean post-intervention (one year later) scores of the emotional scale were significantly lower than pre-intervention scores in both age groups (60 to 74 years and 75 years or more) in both males and females.

The mean score in elderly male subjects was significantly higher in the pre-intervention period compared to females; this difference in means disappeared in the post-intervention period. The decrease between the pre-and post-intervention period was larger in males than in females, which showed that elderly male subjects perceived more their restrictions to participation before the intervention.

The mean post-intervention score in the emotional scale was significantly lower compared to the pre-intervention period in the two age groups (60 to 74 years and 75 years or more). The mean decrease between the pre- and post-intervention periods was larger in elderly subjects aged 75 years or more, showing that this group perceived better their restrictions before the intervention, and therefore benefitted more from it.

The mean scores in the social/situational scale in both periods were significant; they were lower after the intervention in males and females. The mean scores in elderly male subjects were higher compared to females. Thus, the mean decrease between the pre- and post-intervention periods was significantly higher in males, who once again perceived more their restrictions to participation, and therefore benefitted more after the phonological intervention.

Other studies have shown that elderly males perceive more their restrictions than elderly females, which is similar to our findings in the present study.^11^

It is thought that elderly males have a better perception of their restrictions because this group - culturally in our society - is often the family provider even when aged; thus, restrictions to participation are perceived as a handicap.

A few authors have found that benefits of hearing aids use may be evaluated after six months of effective use.^12^ For some authors, however, there were no significant benefits from hearing aids used for less than three months; thus, short intervals between questionnaires would not show the benefits of hearing aids^13^.

It is thought that adaptation time could have an influence on benefits; the reevaluation interval in the present study was one year, which was considered sufficient time to reapply the questionnaire.

Our findings showed that self-awareness of restrictions to participation in elderly subjects decreases after one year of hearing aid use. Such improvement may be attributed only to hearing aid fitting, as reported in several papers. On the other hand, it is important to underline that we monitored our subjects every two months in meetings in which they were able to clear their doubts about hearing aids, receive instructions about communication strategies, and mainly generate affective bonds - a relationship of trust - with the researcher. This procedure undoubtedly influenced the positive outcome of this study.

Among the results of the SF 36 questionnaire, in which higher scores equate with better quality of life, the mean functional ability scores after the intervention were significantly higher irrespective of sex and age, compared to the pre-intervention period.

It should be pointed out that the number (N) of elderly subjects was about half of the total N (50) when the analyses were made according to the variables sex and age group. This reduction may have contributed to the observed lack of significance.

The SF 36 questionnaire showed that the highest number of clinical correlations and statistically significant results among the data was found in the functional ability aspect^7^.

The mean physical aspect scores after the intervention were significantly higher compared to the pre-intervention scores. Our analysis showed that the activities included in the physical aspect improved significantly in the context of general data; they were not affected by the variables sex and age group. This was also observed in the functional ability aspect.

The worse scores in a study that applied the WHOQOL-bref questionnaire were found in the physical domain irrespective of sex. The authors concluded that there were no relationships between hearing loss and the quality of life of elderly subjects in their sample. They found a correlation between age and social relationships, in which more advanced age correlated with less participation in social activities^14^.

The lowest scores in the present study were found in the physical aspect, which was associated with the ability of subjects to perform physical activities such as climbing stairs, walking, and carrying out household chores.

The results of the pain aspect revealed no differences in the mean scores before and after the intervention, irrespective of sex and age group. The mean scores for the age group aged 75 years or more were significantly higher, showing that their quality of life was better compared to the 60 to 74 year age group irrespective of sex and pre-and post-intervention periods. The elderly subjects in the more advanced age group were more active; their energy and well-being were probably the result of better physical status and less pain, which could explain these findings.

The results according to age revealed that subjects at more advanced age had the best scores. According to the authors, this showed that older subjects had a better quality of life. These results also showed that the SF 36 questionnaire was an adequate tool for assessing the quality of life of retired individuals in the study^15^. Results according to age are similar to our finding that subjects at more advanced age (75 years or more) had better scores, therefore better quality of life.

The mean pre- and post-intervention scores of the general health status did not differ; these results were independent of sex and age group. This was expected, as elderly subjects are undergoing a natural aging process.

The results of the physical aspect, pain, and general health status were the worst. In the variable sex, male elderly subjects had better scores in nearly all aspects except for general health status and emotional aspect. In our study, elderly female subjects considered their health worse compared to elderly male subjects^16^. Our results are similar to these findings in that elderly male subjects score better than elderly female subjects.

The vitality aspect scores were significantly higher in the post-intervention period compared to the pre-intervention period irrespective of sex and age group. This is evidence that effective hearing aid use helped improve the quality of life, which was evidenced as better vitality. How do we explain increased vitality associated with hearing amplification? Amplification may foster interaction and better communication, which translates into more energy for activities of daily life.

The elderly subjects performed better in the social aspect after the intervention. The mean post-intervention scores were significantly higher than before the intervention; this result was independent of sex. The difference in mean scores before and after the intervention was age-dependent; it was significantly higher in the 60 to 74 year age group. In the 75 year age group there were no differences in the means of both periods.

Several studies have investigated the relationship between hearing loss and quality of life by using the WHOQOL-bref self-assessment tool; the physical, psychological, social and environmental relationships were assessed. The best scores were seen in the social relationship domain. This was a surprise for researchers, who thought that aging would be a time for social withdrawal, and that the elderly subjects presented hearing loss^16^. In the present study, elderly subjects significantly improved their social aspect scores after the phonoaudiological intervention.

The mean emotional aspect scores were significantly higher after the intervention compared to the pre-intervention period irrespective of sex and age group. Our results showed that the mean score was higher in females aged 75 years or more compared to those aged 60 to 74 years; this result indicated that females are more sensitive to emotional issues. There were no age-related differences in self-awareness of quality of life among male subjects.

Effective use of hearing aids improved communication, which made it possible for elderly individuals to reassume their family and social interactions, thereby improving their quality of life. The author found that the quality of life improved after hearing aids were fitted, especially in the psychological domain, which underlines the importance of hearing - and interventions on hearing - for quality of life and general health in the elderly. The results in the psychological domain improved significantly in both males and females; thus, the variable sex did not affect the results^16^.

The emotional aspect scored low in the present study; the worse results were seen in elderly females, indicating that these individuals were more susceptible to emotional issues.

The mean scores for the mental health aspect were significantly higher after the intervention compared to the pre-intervention period irrespective of sex and age group.

The mean scores in the post-intervention period were higher than in the pre-intervention period in six of eight aspects in the general population (total N), which shows that quality of life improved after the phonoaudiological intervention. Sex and age group differences were in general not seen, which may be explained by the small sample size. On the other hand, analysis of the quality of life by the SF 36 showed that this type of study is relevant for elderly subjects with hearing loss; although it did not include specific questions on communication and hearing, it was sensitive enough to identify an improvement in the quality of life because of amplification. Only two of eight aspects did not improve after one year of hearing aid use and monitoring, namely pain and general health status. This may be explained by the fact that elderly persons experience a chronic condition that gradually restricts their activities of daily life. The aspect vitality improved significantly after one year of hearing aid amplification. This improvement may be attributed to an increased will to participate in activities, as elderly individuals feel safer and become more confident simply by being able to listen and understand speech.

Because the study sample comprised elderly aging subjects with hearing loss and some degree of awareness of their restrictions, the results were both important and expected. The subjects underwent a phonoaudiological intervention - fitting hearing aids - and were monitored during one year.

In the present study, the assessment protocol consisted of self-assessment questionnaires; the results showed that these questionnaires adequately measured changes in quality of life and restrictions to participation in daily life. The HHIE questionnaire, which specifically evaluates hearing, has been used frequently by researchers in this area of study. We found no references in the literature about the use of the SF 36 questionnaire - which evaluates health in general - for assessing the quality of life of individuals with hearing loss. Thus, the results were both surprising and expected; it was possible to measure quality of life improvements and a reduction in restrictions to participation because of a phonoaudiological intervention.

## CONCLUSION

A critical analysis of the results yielded the following conclusions: there is less self-awareness of restrictions to participation after the phonoaudiological intervention in the social and emotional scales in the HHIE questionnaire. Elderly male subjects were more self-aware of restrictions to participation in the emotional and social scales before the phonological intervention. Elderly subjects in the older age group were more self-aware about restrictions to participation in the emotional scale before the phonoaudiological intervention.

The quality of life was shown to improve after the phonoaudiological intervention in the functional ability, physical ability, vitality, emotional, social, and mental health aspects of the SF36 questionnaire irrespective of sex and age.
